# Comprehensive Analysis of lncRNA and miRNA Regulatory Network Reveals Potential Prognostic Non-coding RNA Involved in Breast Cancer Progression

**DOI:** 10.3389/fgene.2021.621809

**Published:** 2021-06-18

**Authors:** Sheng Gao, Xun Lu, Jingjing Ma, Qian Zhou, RanRan Tang, Ziyi Fu, Fengliang Wang, Mingming Lv, Cheng Lu

**Affiliations:** ^1^The First Clinical Medicine College, Nanjing University of Chinese Medicine, Nanjing, China; ^2^Department of Breast, Nanjing Maternity and Child Health Care Hospital, Women’s Hospital of Nanjing Medical University, Nanjing, China; ^3^Milken School of Public Health, George Washington University, Washington, DC, United States; ^4^Nanjing Maternal and Child Health Institute, Nanjing Maternity and Child Health Care Hospital, Women’s Hospital of Nanjing Medical University, Nanjing, China

**Keywords:** breast cancer, microRNA, long non-coding RNA, series test of cluster, overall survival

## Abstract

Breast cancer is one of the most common malignant tumors in women and is the second leading cause of cancer deaths among women. The tumorigenesis and progression of breast cancer are not well understood. The existing researches have indicated that non-coding RNAs, which mainly include long non-coding RNA (lncRNA) and microRNA (miRNA), have gradually become important regulators of breast cancer. We aimed to screen the differential expression of miRNA and lncRNA in the different breast cancer stages and identify the key non-coding RNA using TCGA data. Based on series test of cluster (STC) analysis, bioinformatics analysis, and negatively correlated relationships, 122 lncRNAs, 67 miRNAs, and 119 mRNAs were selected to construct the regulatory network of lncRNA and miRNA. It was shown that the miR-93/20b/106a/106b family was at the center of the regulatory network. Furthermore, 6 miRNAs, 10 lncRNAs, and 15 mRNAs were significantly associated with the overall survival (OS, log-rank *P* < 0.05) of patients with breast cancer. Overexpressed miR-93 in MCF-7 breast cancer cells was associated with suppressed expression of multiple lncRNAs, and these downregulated lncRNAs (MESTIT1, LOC100128164, and DNMBP-AS1) were significantly associated with poor overall survival in breast cancer patients. Therefore, the miR-93/20b/106a/106b family at the core of the regulatory network discovered by our analysis above may be extremely important for the regulation of lncRNA expression and the progression of breast cancer. The identified key miRNA and lncRNA will enhance the understanding of molecular mechanisms of breast cancer progression. Targeting these key non-coding RNA may provide new therapeutic strategies for breast cancer treatment and may prevent the progression of breast cancer from an early stage to an advanced stage.

## Introduction

Breast cancer is one of the most common malignant tumors in women and is the primary reason of cancer deaths among women around the world after lung cancer (Siegel et al., 2018). The 5-year survival rate of patients with early local breast cancer is over 90%, while that of patients with metastatic advanced breast cancer is only 27% (DeSantis et al., 2017), indicating that breast cancer progression, with the formation of distant advanced metastasis, accounts for this poor prognosis. Consequently, the breast cancer patients’ mortality rate is mainly attributed by the advanced patients. Intensive efforts have been made to deepen our understanding of the initiation and progression of breast cancer. However, the molecular mechanisms underlying the tumorigenesis and progression of breast cancer are still not well understood. In particular, it is important to determine how to effectively monitor the progression of the breast cancer from early to late stage.

Most factors involved in the progress of breast cancer are related to changes in the expression of specific genes. Previous researches have indicated that non-coding RNA is an important regulator of gene expression (An and Song, 2011; Aalto and Pasquinelli, 2012). Long non-coding RNA (lncRNA) and microRNA (miRNA) are the two most important regulators (Taft et al., 2010; Chan and Tay, 2018). MiRNA, a small non-coding RNA, is approximately 21–24 nucleotides long and mostly repress the mRNA expression efficiently by base-pairing to the 3′UTR (Finnegan and Pasquinelli, 2013). It has been demonstrated that miRNA associated with proliferation (Johnnidis et al., 2008), differentiation (Becker et al., 2018), cell development (Saultz et al., 2018), and apoptosis (Xu et al., 2016) is linked to all stages of cancers (Gibori et al., 2018; Karimi Mazraehshah et al., 2018; Sun et al., 2018). LncRNA represents an expanding class of ncRNA, consisting of all ncRNA longer than 200 nucleotides (Ponting et al., 2009; Blythe et al., 2016). It has been proved that abnormal expression of lncRNA was related to human diseases, including cancers. Several specific miRNA and lncRNA were associated with the different stages of breast cancer and hold great promise as biomarkers and therapeutic targets (Wang et al., 2017; Adhami et al., 2018).

Non-coding RNAs show a time- or tissue-specific expression pattern and play important roles in a variety of biological processes (Derrien et al., 2012; Li et al., 2015). Therefore, they may display different patterns in breast cancer development and progression. The tumor-node-metastasis classification (TNM classification) of malignant tumors is the most common system used to evaluate the treatment and prognosis of cancer and represents different stages of cancer progression (Greene, 2004; Singletary and Connolly, 2006).

In the present study, differentially expressed miRNA and lncRNA of breast cancer different stages were acquired by the Cancer Genome Atlas (TCGA). TCGA, a landmark cancer genomics program, molecularly characterized over 20,000 primary cancer and matched normal samples spanning 33 cancer types, with the goal of improving the understanding of the molecular basis of cancer and advancing the ability to diagnose, treat, and prevent cancer. Based on series test of cluster (STC) analysis, the expression patterns of miRNA/lncRNA were identified in breast cancer progression. We also constructed the regulatory network of lncRNA and miRNA to determine the key lncRNA and miRNA in breast cancer progression. Finally, the potential prognostic miRNA, lncRNA, and mRNA involved in breast cancer were revealed. The identified key lncRNA and miRNA in our work would enhance the understanding of mechanism of progression of breast cancer, which may serve as the potential therapeutic targets.

## Materials and Methods

### RNA Expression Data and Sample Grouping

The RNA expression data and clinical information of breast cancer patients were collected from the TCGA database. Patients with the following conditions are excluded: (i) patients suffering from other malignancies besides breast cancer; (ii) male breast cancer cases; (iii) patients without complete staging information; and (iv) overall survival (OS) > 2,000 days. To seek the abnormally expressed miRNA/lncRNA in the different stages of breast cancer, all patients in TCGA were eventually enrolled in stage I, stage II, and stage III and IV groups.

### Screening of Differentially Expressed Non-coding RNA

**FIGURE 1:**
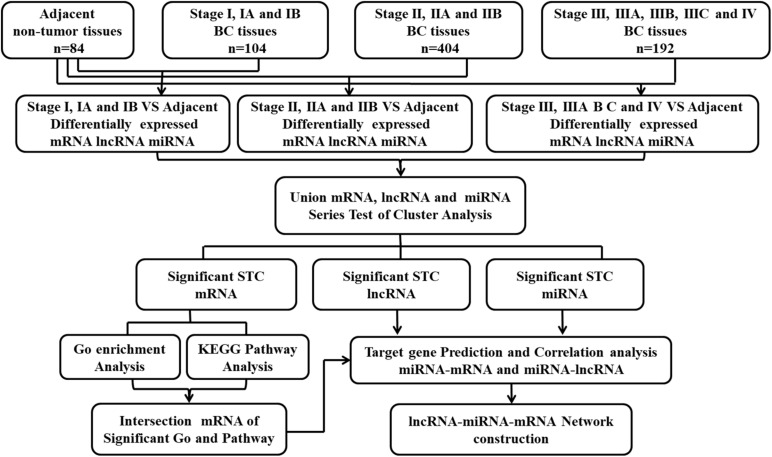
Flow-chart of data analysis.

Eighty-four adjacent non-tumor tissues were set as normal controls. The differentially expressed RNA between the adjacent normal tissues and stage I, stage II, and stage III and IV cancer tissues was screened. The process of screening deregulated genes is shown in Figure 1. The differentially expressed non-coding RNAs were also screened between the normal tissues and cancer tissues concluding all the stages.

### STC Analysis

The experiment was carried out on the basis of previous studies (Luan et al., 2017). When the diseases progressed from one stage to the next or when organisms were subjected to environmental stimulation, the gene expression changes exhibited different trend characteristics. STC analysis could reveal the gene expression’s trend characteristics. The most representative genes with the same change characteristic could be confirmed in the disease progression and were gathered into the different changing-trend profile. We set adjacent non-tumor tissues, stages I, II, and III and IV, as different points of breast cancer development. The differential expression genes at a logical sequence according to RVM (random variance model) corrective ANOVA were selected. The raw expression values were converted into log2 ratio. The expression model profiles are related to the actual or the expected number of genes assigned to each model profile. Significant profiles have higher probability than expected by Fisher’s exact test and multiple comparison test.

### Bioinformatic Analysis of Differentially Expressed Genes

The Gene Ontology database and KEGG (Kyoto Encyclopedia of Genes and Genomes) were used to analyze the functions of the differential expression of genes in significant patterns of the STC analysis. Two-sided Fisher’s exact test and χ^2^ test were used to classify GO enrichment and select the significant KEGG pathway, and FDR (false discovery rate) was calculated to correct the *p*-value. Genes that were upregulated and downregulated were analyzed. We were interested in GO biological processes and KEGG pathways with statistical significance. FDR < 0.05 was considered statistically significant.

### Construction of lncRNA and miRNA Regulatory Network

LncRNA can act as a miRNA decoy or sponge by complementary sequence and can indirectly regulate the expression and activity of mRNA. Based on this theory, the lncRNA and miRNA regulatory network was constructed. The differentially expressed miRNAs with significant patterns from the STC analysis were selected. MRNAs targeted by these miRNAs were predicted by TargetScan and miRanda. The predicted lncRNA targeted by these miRNAs were determined by miRanda. Then, we combined predicted lncRNA/mRNA and the differentially expressed lncRNA/mRNA to choose the intersecting lncRNA and mRNA. Finally, according to the theory that miRNA negatively regulates lncRNA and mRNA, the miRNA–lncRNA and miRNA–mRNA relationships of negative correlation were screened, and the lncRNA and miRNA regulatory network was constructed.

### Cell Culture and Transfection

Human breast cancer cell line MCF-7 was purchased from American Type Culture Collection and maintained in RPMI 1640 containing 10% FBS and 1% penicillin/streptomycin (Gibico) in a humidified atmosphere of 5% CO2 at 37°C. Cells were transfected with mimic/inhibitor for miR-93-5p or control (RiboBio) mixed with Opti-MEM medium using Lipofectamine 2000 (Invitrogen).

### Total RNA Extraction and Real-Time PCR

MCF-7 cells were transiently transfected with miR-93-5p mimic/inhibitor or control, and after 48 h of transfection, total RNA was isolated using TRIzol reagent (Invitrogen). cDNA was synthesized using oligodT primers or miR-93-5p/U6-specific primers (RiboBio) with a Reverse transcription kit (Thermo fisher). Real-time PCR was performed in triplicate using TaqMan Universal PCR Master Mix (Applied Biosystems) in the ViiA7 system (Applied Biosystems). The primers of miRNA-93-5P were designed and provided by RiboBio.

### Statistical Analysis

The associations between breast cancer patients’ overall survival time and the specific lncRNA/mRNA expression levels were analyzed by using Kaplan–Meier method, the Cox proportional hazard regression models, and log-rank test.

## Results

### Differential Expression of miRNA and lncRNA in the Different Stages

The mRNA, miRNA, and lncRNA expression data of 1,097 breast cancer from TCGA were collected and obtained. According to the clinical information given in the TCGA database, we removed 397 unsuitable cases, and 700 cases were included in this study. To seek the deregulated lncRNA and miRNA in the different stages of breast cancer, 700 cases in TCGA were divided into three groups (stage I, stage II, and stages III and IV) according to their diagnosed stages that were based on the recommendations of the AJCC. There were 104 cases presented with stage I, 404 cases presented with stage II, and 192 cases presented with stages III and IV. In addition, 84 adjacent non-tumor tissues were set as normal controls to screen the differentially expressed RNA. The process of screening the deregulated genes and that of the bioinformatics analysis is shown in Figure 1. We identified differentially expressed 193 lncRNA and 148 miRNA between stage I and the adjacent normal tissues, 208 lncRNA and 166 miRNA between stage II and the adjacent normal tissues, 207 lncRNA and 163 miRNA between stages III and IV, and the adjacent normal tissues (absolute fold change > 2, FDR < 0.05, and *P* < 0.05). In addition, we identified differentially expressed 204 lncRNA and 162 miRNA between the normal tissues and cancer tissues concluding all the stages (Supplementary Table 1).

### MiRNA/LncRNA Expression Model Analysis With STC Analysis

STC is used to reveal the expression tendency of the genes. To further assess the profile pattern of differentially expressed miRNA/lncRNA among the different stage of breast cancer, we used the union of differentially expressed miRNA/lncRNA in the breast cancer different stage, and STC analysis was performed. There were 26 possible model (Supplementary Figure 1) profiles to enrich the expression tendency of the miRNA/lncRNA. We identified five expression patterns of aberrantly expressed miRNA (profiles 2, 4, 5, 22, and 25) and five expression patterns of aberrantly expressed lncRNA (profiles 1, 2, 5, 22, and 25) that were significant (*P* < 0.05, Supplementary Figure 2). The differential expression of 224 lncRNA and 172 miRNA in the significantly changing trend profiles might have vital roles in breast cancer progression. We also identified eight patterns of aberrantly expressed mRNA (profiles 1, 2, 4, 5, 7, 22, 25, and 26) that were significant (*P* < 0.05) for the following analysis.

### GO Enrichment and KEGG Pathway Analysis

**FIGURE 2:**
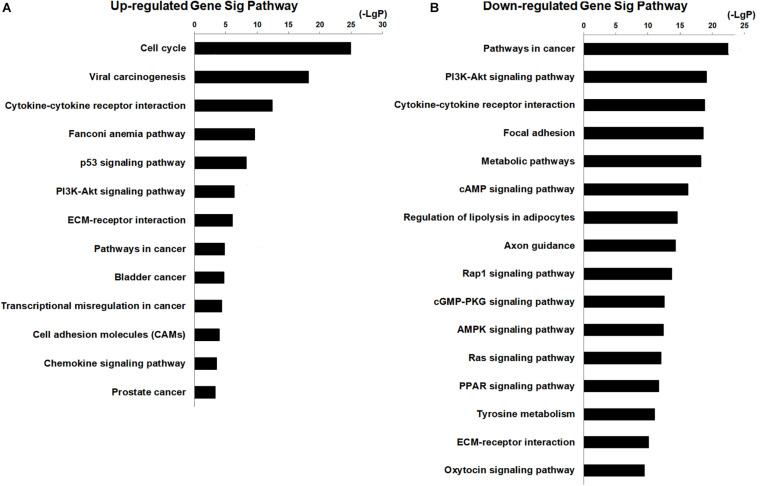
KEGG pathway analysis for aberrantly expressed mRNAs in the progression of breast cancer. **(A)** Thirteen significant pathways in the top 20 upregulated genes. **(B)** Sixteen pathways in the top 20 downregulated genes.

The potential functions of non-coding RNA were identified by GO and KEGG pathways analysis. There were 17 terms related to breast cancer in the top 20 GO analysis terms (upregulated genes), and 8 of the terms included cell division, cell proliferation, cell cycle, and DNA repair (Supplementary Figure 3). There were 15 terms related to cancer in the top 20 GO analysis terms (downregulated genes), and 9 of the terms included signal transduction, cell adhesion, angiogenesis, apoptosis, and cell proliferation (Supplementary Figure 3). These GO analyses indicated that aberrantly expressed genes were mostly related to cell proliferation, cell apoptosis, cell cycle, cell adhesion, and angiogenesis, which were related to the biological behavior of cancer cells. Pathway analysis indicated that upregulated genes are related to 63 pathways and that there were 13 pathways in the top 20 pathways related to breast cancer (Figure 2A). The downregulated genes are enriched in 144 pathways, and there were 16 pathways in the top 20 pathways related to cancer (Figure 2B). The potential signaling pathways have been identified in breast cancer progression, including cell cycle pathway, P53 signaling pathway, PI3K–AKT signaling pathways, focal adhesion pathways, pathways in cancer, metabolic pathway, cAMP pathway, AMPK signal pathway, and Ras signal pathway (Figure 2). These findings suggested that the aberrant expression genes were mainly enriched in cancer cells’ biological behavior. At the same time, the results also suggested that downregulated genes were concerned with the regulation of miRNA in cancer. To further narrow the possible target genes, we combined the genes in the GO and Pathway analysis and chose 637 intersecting genes with significant difference (FDR < 0.05) to perform the following analysis.

### Prediction of the miRNA Targets and Construction of the lncRNA and miRNA Regulatory Network

**FIGURE 3:**
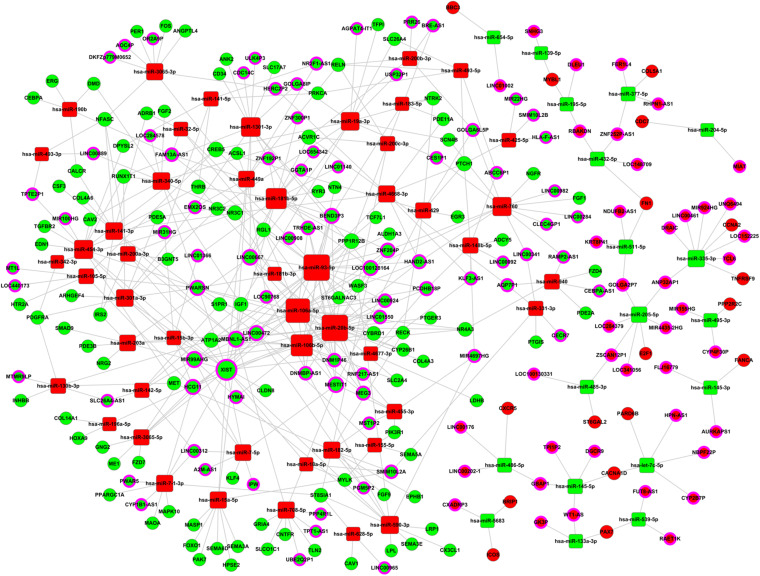
LncRNA–miRNA–mRNA regulatory network. Squares represent miRNAs, balls represent mRNAs, and balls with a circle around represent lncRNAs. Red means upregulated genes, while green means downregulated genes.

Firstly, 172 differentially expressed miRNA (in five significant patterns in the above STC analysis) were selected to further predict their targeted mRNA by miRanda and TargetScan. MiRNA may interact with lncRNA through miRNA recognition elements. We predicted the lncRNA targeted by these miRNAs by miRanda, and the results show that these 156 miRNAs target 200 lncRNAs. Then, we chose the intersecting lncRNA and mRNA between the predicted target mRNA/lncRNA, and we chose differential expression of 220 lncRNA and 637 mRNA from above. Finally, we selected these differentially expressed RNAs that were negatively regulated between the lncRNA/mRNA and the miRNA according to the theory that miRNA negatively regulates lncRNA and mRNA. Based on the above data, 122 lncRNAs, 67 miRNAs, and 119 mRNAs were selected to construct the regulatory network of lncRNA and miRNA (Figure 3). Degree refers to the degree of association or connectivity between genes and other genes in gene interaction network, which is the most basic characteristic of a gene-to-gene interaction. The genes with the highest degree value in gene interaction network have the greatest influence on the overall structure of the network and other gene relationships. The top 15 key lncRNAs and miRNAs that may target these lncRNA are shown in Supplementary Table 2. The top 15 key miRNA and their target lncRNA/mRNA in the regulatory network in breast cancer progression are shown in Supplementary Table 3. We found that in the regulatory network, miR-301a-3p (Zheng et al., 2018), miR-93-5p (Ni et al., 2018), miR-454-3p (Ren et al., 2019), miR-181b-5p (Sochor et al., 2014), XIST (Salvador et al., 2013), LINC00472 (Wang et al., 2019), MIR31HG (Augoff et al., 2012), MEG3 (Mondal et al., 2015), MIR155HG (Ghafouri-Fard et al., 2020), and LINC00667 (Zhu et al., 2020) have been reported in breast cancer. This indicates that our results are consistent with the results of existing research. Some non-coding RNAs that have not been reported in breast cancer need further research to reveal their functions and mechanisms. It is worth noting that the miR-93/20b/106a/106b family is located at the center of the regulatory network.

### Potential Biological Functions of the Aberrantly Expressed miR-93/20b/106a/106b Family in Breast Cancer

**FIGURE 4:**
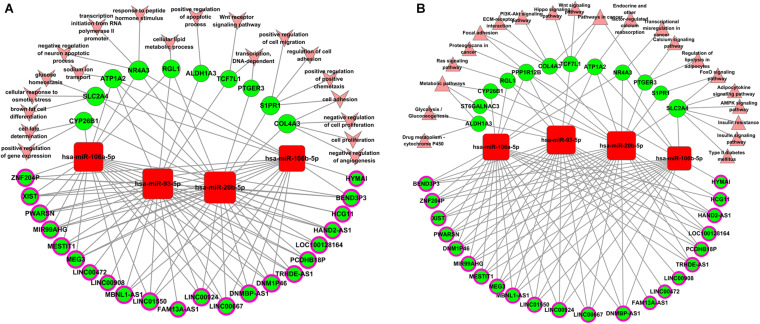
Biological function analysis of aberrantly expressed genes regulated by the miR-93/20b/106a/106b family in breast cancer. **(A)** Gene ontology terms analysis. **(B)** Pathways enrichment analysis. Squares represent miRNAs, balls represent mRNAs, and balls with a circle around represent lncRNAs. Red means upregulated genes, while green means downregulated genes. Arrows represent the GO terms, and triangles represent the pathway terms.

MiR-106b, miR-106a, miR-93, and miR-20b share the same seed sequence “AAAGUGC,” which is encoded in two genetic clusters and has similar physiological functions in a variety of diseases (Supplementary Figure 4A). They all show a profile 22 pattern in the five aberrantly expressed patterns of miRNA (Supplementary Figure 4B). MiR-20b and miR-106a are encoded by the miR-106a-363 cluster located in chromosome X. MiR-93 and miR-106b were encoded by the miR-106b-25 cluster located in chromosome 7. The relative expression of miR-93 was the highest (Supplementary Figure 4C). In view of the importance of the miRNA-93/20b/106a/106b family and the related lncRNA, which were separately displayed, further GO and KEGG pathway analyses were conducted. As shown in Table 1, all 22 lncRNAs and 14 mRNAs in the miRNA-93/20b/106a/106b family are in the regulatory network. The GO and KEGG pathway analyses show that the miR-93/20b/106a/106b family members are mainly involved in development, ion transport, and metabolism. The go and pathway terms that related to cancer were displayed in Figure 4.

**TABLE 1:** miRNA-93/20b/106a/106b family and related lncRNAs/mRNAs in lncRNA-miRNA-mRNA regulatory network.

miRNA	LncRNA	mRNA
miR-20b-5p miR-93-5p miR-106a-5p mir-106b-5p	BEND3P3, DNM1P46, DNMBP-AS1, HAND2-AS1, HCG11, LINC00667, LINC00924, LINC01550, LOC100128164, MBNL1-AS1, MEG3, MESTIT1, MIR99AHG, PCDHB18P, PWARSN, TRHDE-AS1, XIST, ZNF204P, FAM13A-AS1, LINC00472, LINC00908, LINC00924	ATP1A2, COL4A3, CYBRD1, CYP26B1, NR4A3, PPP1R12B, PTGER3, RGL1, S1PR1, SLC2A4,ALDH1A3, NTN4, ST6GALNAC3, TCF7L1

### Survival Prediction of Non-coding RNA and mRNA

**FIGURE 5:**
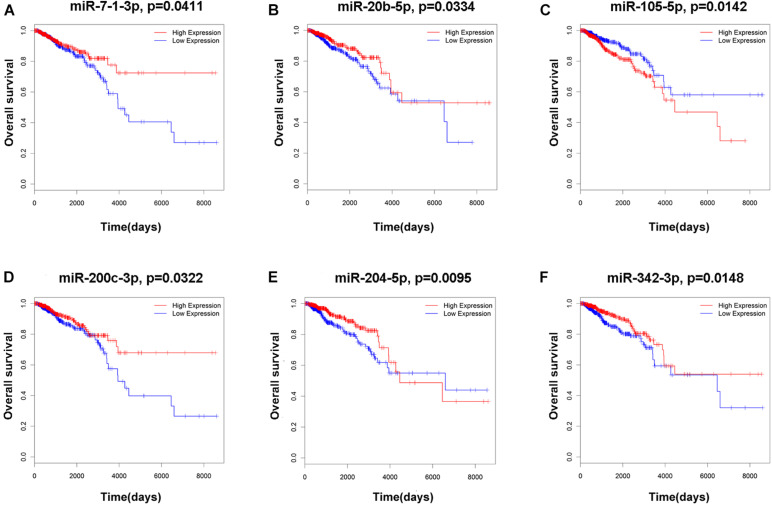
Kaplan–Meier survival curves for six miRNAs associated with overall survival. **(A)** miR-7-1-3p. **(B)** miR-20b-5p. **(C)** miR-105-5p. **(D)** miR-200c-3p. **(E)** miR-204-5p. **(F)** miR-342-3p.

**FIGURE 6:**
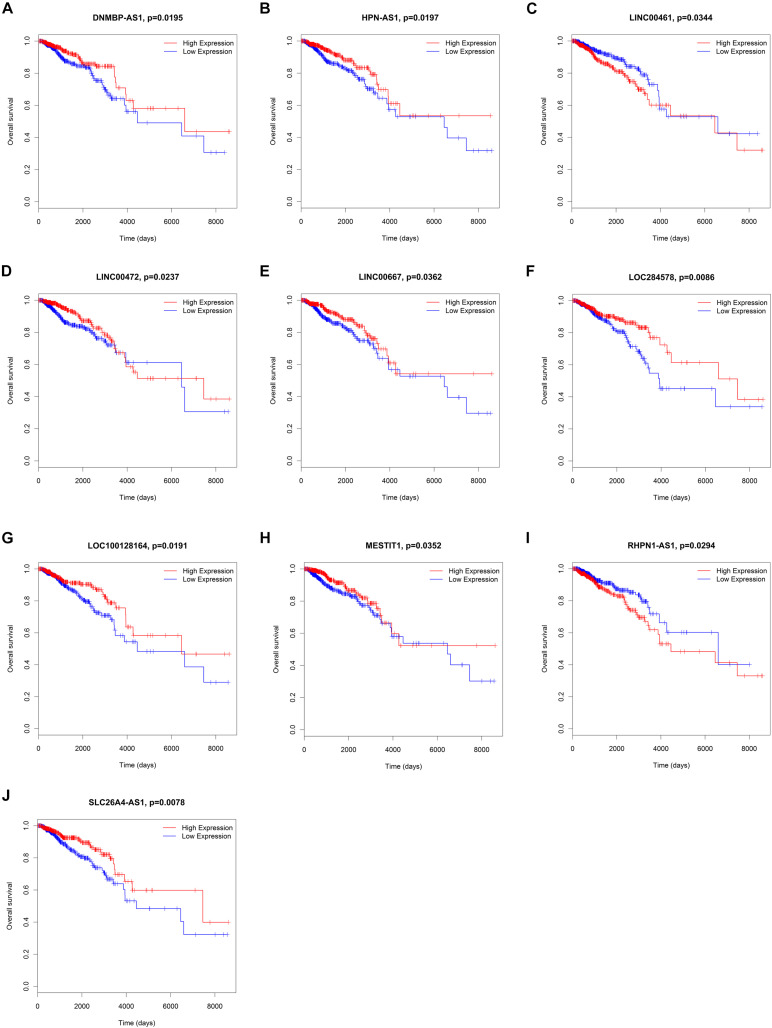
Kaplan–Meier survival curves for 10 lncRNAs associated with overall survival. **(A)** DNMBP-AS1. **(B)** HPN-AS1. **(C)** LINC00461. **(D)** LINC00472. **(E)** LINC00667. **(F)** LOC284578. **(G)** LOC100128164. **(H)** MESTIT1. **(I)** RHPN1-AS1. **(J)** SLC26A4-AS1.

**FIGURE 7:**
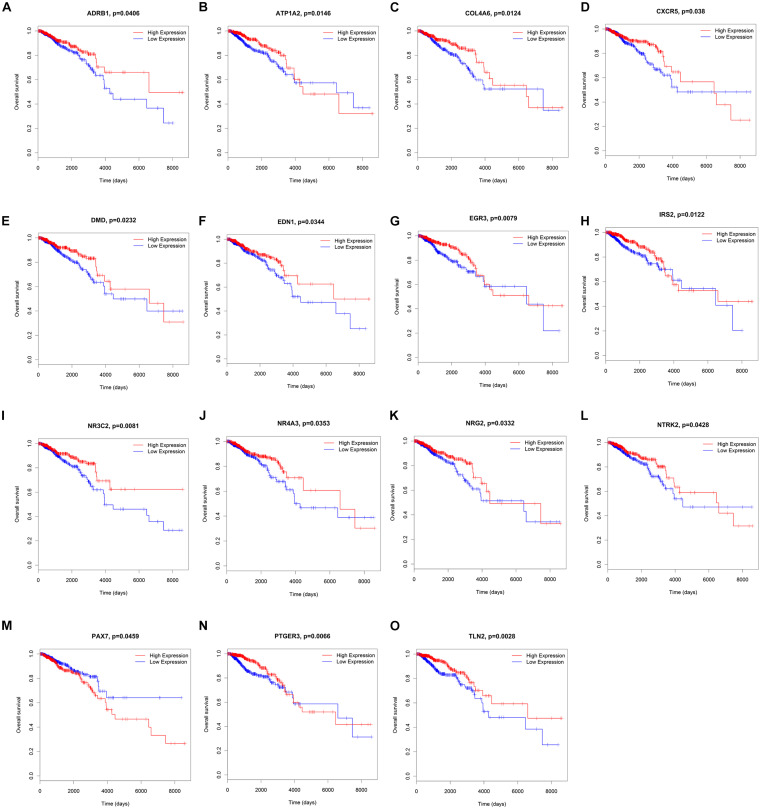
Kaplan–Meier survival curves for 15 mRNAs associated with overall survival. **(A)** ADRB1, **(B)** ATP1A2, **(C)** COL4A6, **(D)** CXCR5, **(E)** DMD, **(F)** EDN1, **(G)** EGR3, **(H)** IRS2, **(I)** NR3C2, **(J)** NR4A3, **(K)** NRG2, **(L)** NTRK2, **(M)** PAX7, **(N)** PTGER3, **(O)** TLN2.

To elucidate the relationship between these key genes (non-coding RNA and mRNA) and the outcome of breast cancer patients, univariate Cox proportional hazards regression model was used to validate their prognostic values. MiR-200c-3p, miR-204-5p, miR-20b-5p, miR-7-1-3p, miR-105-5p, miR-342-3p, DNMBP-AS1, HPN-AS1, LINC00461, LINC00472, LINC00667, LOC100128164, LOC284578, MESTIT1, RHPN1-AS1, and SLC26A4-AS1 were significantly associated with breast cancer patients’ overall survival (log-rank *P* < 0.05, Figures 5, 6). Additionally, we found 15 mRNAs that were significantly related to overall survival (Figure 7). The expression of these 6 miRNAs, 10 lncRNAs, and 15 mRNAs is shown in Table 2. In addition, the relationship between these miRNA, lncRNA, and mRNA expression levels and prognosis in breast cancer patients is also shown in Table 2. CXCR5, miR-200c-3p, miR-20b-5p, miR-7-1-3p, miR-342-3p, and HPN-AS1 were upregulated genes, while these high expression genes were significantly associated with breast cancer patients’ better overall survival. Among these non-coding RNAs, miR-20b-5p, MESTIT1, LOC100128164, LINC00667, and DNMBP-AS1 were related to the miR-93/20b/106a/106b family.

**TABLE 2:** lncRNA/miRNA/mRNAs that were significantly related to overall survival in breast cancer progression.

Gene_id	Gene type	Expression	Hazard ratio	*p*-Value	Relationship to overall survival
DMD	mRNA	Down	0.6371	0.0232	Positive
NRG2	mRNA	Down	0.6567	0.0332	Positive
NTRK2	mRNA	Down	0.6715	0.0428	Positive
PAX7	mRNA	Up	1.4916	0.0459	Negative
PTGER3	mRNA	Down	0.5840	0.0066	Positive
TLN2	mRNA	Down	0.5486	0.0028	Positive
ADRB1	mRNA	Down	0.6668	0.0406	Positive
ATP1A2	mRNA	Down	0.6134	0.0146	Positive
COL4A6	mRNA	Down	0.6097	0.0124	Positive
CXCR5	mRNA	Up	0.6629	0.0380	Positive
EDN1	mRNA	Down	0.6562	0.0344	Positive
EGR3	mRNA	Down	0.5929	0.0079	Positive
IRS2	mRNA	Down	0.6115	0.0122	Positive
NR3C2	mRNA	Down	0.5871	0.0081	Positive
NR4A3	mRNA	Down	0.6588	0.0353	Positive
miR-200c-3p	miRNA	Up	0.6471	0.0322	Positive
miR-204-5p	miRNA	Down	0.5939	0.0095	Positive
miR-20b-5p	miRNA	Up	0.6506	0.0334	Positive
miR-7-1-3p	miRNA	Up	0.6590	0.0411	Positive
miR-105-5p	miRNA	Up	1.6361	0.0142	Negative
miR-342-3p	miRNA	Up	0.6136	0.0148	Positive
DNMBP-AS1	lncRNA	Down	0.6268	0.0195	Positive
HPN-AS1	lncRNA	Up	0.6263	0.0197	Positive
LINC00461	lncRNA	Up	1.5183	0.0344	Negative
LINC00472	lncRNA	Down	0.6394	0.0237	Positive
LINC00667	lncRNA	Down	0.6600	0.0362	Positive
LOC100128164	lncRNA	Down	0.6267	0.0191	Positive
LOC284578	lncRNA	Down	0.5961	0.0086	Positive
MESTIT1	lncRNA	Down	0.6527	0.0352	Positive
RHPN1-AS1	lncRNA	Up	1.5462	0.0294	Negative
SLC26A4-AS1	lncRNA	Down	0.5895	0.0078	Positive

### Experimental Verification of Possible Regulatory Relationships Between miR-93 and Some lncRNA/mRNA

**FIGURE 8:**
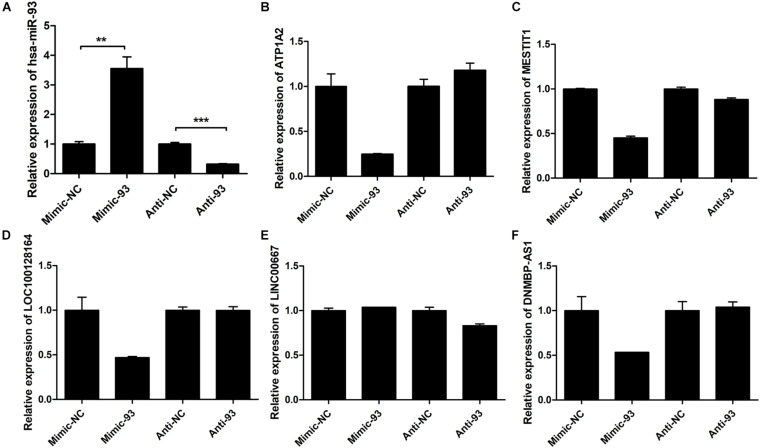
The expression levels of miR-93-5P and some mRNA and lncRNAs. **(A)** Expression of miR-93-5P was examined by qRT-PCR 48 h post-transfection with miR-93-5p mimic/inhibitor or control. U6 gene was used as internal control. **(B)** ATP1A2, **(C)** MESTIT1, **(D)** LOC100128164, **(E)** LINC00667, **(F)** DNMBP-AS1.

Some central lncRNAs and miRNAs predicted here may have vital roles in breast cancer progression. Therefore, we further used GEO data to analyze the expression levels of four miRNAs in the miRNA-93/20b/106a/106b family at the core of the regulatory network. The results showed that miRNA-93/20b/106a/106b were significantly and highly expressed in tumor tissues in one or more GEO data (Supplementary Figure 5). Among them, the expression of miR-93 is the most stable and relatively significant. Next, MCF-7 cells were transiently transfected with miR-93-5p mimic/inhibitor or control to validate the miRNA-93/20b/106a/106b family regulatory network. The expression levels of miR-93-5P were examined by qRT-PCR after transfection with miR-93-5p mimic/inhibitor or control (Figure 8A). It was found that the expression levels of ATP1A2, MESTIT1, LOC100128164, and DNMBP-AS1 were all downregulated by overexpressing miR-93 in MCF-7 cells. Therefore, the miRNA-93/20b/106a/106b family suppresses multiple lncRNA expression (MESTIT1, LOC100128164, and DNMBP-AS1). However, these gene expression levels did not significantly change after treatment with miR-93-5p inhibitor (Figures 8B–F). The above results can indicate that overexpressed miR-93 in breast cancer tissues is associated with the repression of multiple lncRNAs, and these downregulated lncRNAs (MESTIT1, LOC100128164, and DNMBP-AS1) were significantly associated with poor overall survival in breast cancer patients.

In this study, we found low expression mRNAs/lncRNAs were significantly associated with cancer patients’ better overall survival and high expression mRNAs were significantly associated with cancer patients’ worse overall survival except for CXCR5 and HPN-AS1. However, upregulated miRNAs (miR-200c-3p, miR-20b-5p, miR-7-1-3p, and miR-342-3p) were significantly associated with breast cancer patients’ better overall survival. It is certain that some lncRNAs suppressed by the highly expressed miRNA-93/20b/106a/106b family are not conducive to the prognosis of patients, and the regulation between miRNAs and lncRNAs may be more complicated.

## Discussion

Breast cancer is one of the most common malignant tumors in women and is the primary reason of cancer deaths among women around the world. The miRNA and lncRNA expression profiles were analyzed in a variety of human cancer using high-throughput microarrays or sequencing. The biological functions of non-coding RNA in breast cancer have been revealed, including tumorigenesis, proliferation, apoptosis, and invasion. In previous studies, the differentially expressed miRNA and lncRNA were mostly screened from the different subtypes of breast cancer tissues, cancerous and normal breast tissues, and breast cancer drug-resistant cells (Van Schooneveld et al., 2012; Lv et al., 2014; Sun et al., 2014; Chen et al., 2015). Most studies focus on the function and mechanism of single non-coding RNA. Recently, evidence has begun to accumulate that these two ncRNAs can interact with each other and can influence the gene expression in breast cancer (Zhang H.Y. et al., 2017; Lu et al., 2018; Zhao et al., 2018). Other studies have reported that miR-510, PVT1, CCAT1, and Linc00861 may play important roles in breast invasive carcinoma (Zhang Y. et al., 2017). MiR-19a and lncRNA DLEU1 might be co-expressed to regulate the expression of ER in breast cancer (Wu et al., 2015).

In our research, we successfully obtained differentially expressed miRNA and lncRNA in the different stages of breast cancer by bioinformatics analysis based on large numbers of samples and clinical information from the TCGA database. Moreover, we analyzed the abnormal expression patterns of miRNA and lncRNA by STC analysis. A total of 67 miRNAs, 122 lncRNAs, and 119 mRNAs were selected to construct the non-coding regulatory network. The results showed that the miR-93/20b/106a/106b family was in the center of the regulatory network, which may help to determine the key lncRNA and target genes that participated in the different stages of breast cancer. MiR-20b, miR-106a, miR-93, and miR-106b were overexpressed in tumor tissue, serum, or plasma samples from patients with cancer, including breast cancer (Fang et al., 2012; Deng et al., 2014; Zheng et al., 2015; Li M. et al., 2018). It has been demonstrated that the miR-93/20b/106a/106b family plays important roles in breast cancer and other cancers by regulating their target genes. Notch, MAPK, PI3K/Akt, and Hippo pathways are also involved in the regulation of miR-93/20b/106a/106b family (Shen et al., 2016; Li et al., 2017; Guarnieri et al., 2018; Li L. et al., 2018).

We also observed that 6 miRNAs (miR-7-1-3p, miR-20b-5p, miR-105-5p, miR-200c-3p, miR-204-5p, and miR-342-3p) and 10 lncRNA (DNMBP-AS1, HPN-AS1, LINC00461, LINC00472, LINC00667, LOC100128164, LOC284578, MESTIT1, RHPN1-AS1, and SLC26A4-AS1) can predict overall survival of patients with breast cancer. In existing research, highly expressed genes were significantly associated with cancer patients’ worse overall survival, and genes with low expression were significantly associated with better overall survival. In this study, we found that upregulated miRNAs (miR-200c-3p, miR-20b-5p, miR-7-1-3p, and miR-342-3p) were significantly associated with breast cancer patients’ better overall survival.

Previous findings also showed that miRNA was a universal mechanism of post-transcriptional gene regulation that controlled precise gene expression. MiRNA is the regulator of gene expression, and accumulating evidence has suggested that miRNAs share a key role in breast cancer progression through the regulation of their target genes. More importantly, a single miRNA may regulate multiple target genes (Buchan and Parker, 2007; Wu and Pfeffer, 2016); therefore, multiple target genes jointly determine the function of miRNA in cancer patients’ overall survival. For these reasons, some high expression of miRNAs is associated with breast cancer patients’ better overall survival. Besides, compared to mRNA and lncRNA, the regulation of miRNA expression is complicated. During transcription, miRNAs were transcribed by RNA Polymerase II as long primary transcripts characterized by hairpin structures (pri-miRNAs). After transcription, the enzymes Drosha and Dicer process pri-miRNAs and pre-miRNAs to generate mature miRNAs (Feng et al., 2011; Iorio and Croce, 2012). Therefore, our results suggest that lncRNA/mRNA may be more suitable as prognostic biomarkers than miRNAs. The paradox of relationship between miRNA expression with breast cancer prognosis may be important in the progression of breast cancer, and it is more important to clarify this phenomenon in the prevention and treatment of breast cancer.

## Conclusion

In conclusion, we screened abnormal expression of miRNA and lncRNA using TCGA data. STC analysis, lncRNA–miRNA–mRNA regulatory network, GEO data, and cell experiments showed that key miRNA and lncRNA performed vital roles in breast cancer progression. However, these non-coding RNA related functions and mechanisms still need to be further developed, especially these miRNA and lncRNA with prognostic value. Targeting these key non-coding RNAs may provide new therapeutic options for patients with breast cancer and may prevent the development of breast cancer from the early stages to an advanced disease.

## Data Availability Statement

Publicly available datasets were analyzed in this study. This data can be found here: https://www.cancer.gov/about-nci/organization/ccg/research/structural-genomics/tcga.

## Author Contributions

ML and CL constructed the main conceptual ideas and proposed the research. SG, XL, JM, and QZ conceived the technical details, performed the analysis, and prepared the manuscript. RT, ZF, and FW collected and processed the data. All authors contributed to the article and approved the submitted version.

## Conflict of Interest

The authors declare that the research was conducted in the absence of any commercial or financial relationships that could be construed as a potential conflict of interest.

## Abbreviations

TCGA: the Cancer Genome Atlas
OS: overall survival
STC: series test of cluster
AJCC: American Joint Committee on Cancer.
