# Supplementary material for: Comprehensive Analysis of lncRNA and miRNA Regulatory Network Reveals Potential Prognostic Non-coding RNA Involved in Breast Cancer Progression
Source: Front Genet. 2021 Jun 18;12:621809. doi: 10.3389/fgene.2021.621809 (PMC8253500; doi:10.3389/fgene.2021.621809)
Supplement: Supplementary file 1 [file Data_Sheet_1.zip › Table 2 - 3.DOCX]

Tables

Table S2 Top 15 key lncRNAs and miRNAs that may target these key lncRNAs in breast cancer progression

| **Key LncRNA** | **miRNAs that may target these keylncRNAs** |
| --- | --- |
| XIST | miR-106a-5p, miR-106b-5p, miR-15a-5p, miR-181b-5p, miR-18a-5p, miR-196a-5p, miR-200a-3p, miR-203a, miR-20b-5p, miR-301a-3p, miR-3065-5p, miR-340-5p, miR-449a, miR-454-3p, miR-4677-3p, miR-590-3p, miR-7-1-3p, miR-7-5p, miR-93-5p |
| MBNL1-AS1 | miR-106a-5p, miR-106b-5p, miR-1301-3p, miR-141-3p, miR-200a-3p, miR-203a, miR-20b-5p, miR-708-5p, miR-93-5p |
| MIR99AHG | miR-106a-5p, miR-106b-5p, miR-142-5p, miR-20b-5p, miR-301a-3p, miR-3065-5p, miR-454-3p, miR-7-5p, miR-93-5p |
| LINC00667 | miR-106a-5p, miR-106b-5p, miR-1301-3p, miR-181b-5p, miR-19a-3p, miR-203a, miR-20b-5p, miR-93-5p |
| MESTIT1 | miR-106a-5p, miR-106b-5p, miR-20b-5p, miR-429, miR-455-3p, miR-708-5p, miR-7-5p, miR-93-5p |
| BEND3P3 | miR-106a-5p, miR-106b-5p, miR-1301-3p, miR-181b-5p, miR-183-5p, miR-20b-5p, miR-93-5p |
| HCG11 | miR-105-5p, miR-106a-5p, miR-15b-3p, miR-20b-5p, miR-3065-5p, miR-7-1-3p, miR-7-5p, |
| LINC00472 | miR-141-3p, miR-155-5p, miR-203a, miR-3065-5p, miR-4668-3p, miR-590-3p, miR-93-5p |
| LOC100128164 | miR-106a-5p, miR-1301-3p, miR-181b-5p, miR-182-5p, miR-20b-5p, miR-760, miR-93-5p |
| PWARSN | miR-105-5p, miR-106a-5p, miR-106b-5p, miR-20b-5p, miR-301a-3p, miR-454-3p, miR-93-5p |
| ZNF204P | miR-106a-5p, miR-106b-5p, miR-181b-5p, miR-20b-5p, miR-760, miR-93-5p |
| HAND2-AS1 | miR-106a-5p, miR-20b-5p, miR-4668-3p, miR-760, miR-93-5p |
| MEG3 | miR-106a-5p, miR-106b-5p, miR-20b-5p, miR-455-3p, miR-93-5p |
| PCDHB18P | miR-106a-5p, miR-148b-5p, miR-20b-5p, miR-4677-3p, miR-93-5p |
| DNM1P46 | miR-106a-5p, miR-106b-5p, miR-20b-5p, miR-93-5p |

Table S3 Top 15 key miRNAs and target lnRNAs/mRNA in the regulation network in breast cancer progression

| **miRNA** | **miRNAs targeting lncRNAs** | **miRNAs targeting mRNAs** |
| --- | --- | --- |
| miR-20b-5p | BEND3P3, DNM1P46, DNMBP-AS1, HAND2-AS1, HCG11, LINC00667, LINC00924, LINC01550, LOC100128164, MBNL1-AS1, MEG3, MESTIT1, MIR99AHG, PCDHB18P, PWARSN, TRHDE-AS1, XIST, ZNF204P | ATP1A2, COL4A3, CYBRD1, CYP26B1, NR4A3, PPP1R12B, PTGER3, RGL1, S1PR1, SLC2A4 |
| miR-93-5p | BEND3P3, DNM1P46, DNMBP-AS1, FAM13A-AS1, HAND2-AS1, LINC00472, LINC00667, LINC00908, LINC00924, LINC01550, LOC100128164, MBNL1-AS1, MEG3, MESTIT1, MIR99AHG, PCDHB18P, PWARSN, TRHDE-AS1, XIST, ZNF204P | ALDH1A3, ATP1A2, CYBRD1, NTN4, PPP1R12B, RGL1, ST6GALNAC3, TCF7L1 |
| miR-106a-5p | BEND3P3, DNM1P46, DNMBP-AS1, HAND2-AS1, HCG11, LINC00667, LINC00924, LINC01550, LOC100128164, MBNL1-AS1, MEG3, MESTIT1, MIR99AHG, PCDHB18P, PWARSN, TRHDE-AS1, XIST, ZNF204P | ATP1A2, CYBRD1, CYP26B1, NR4A3, PPP1R12B, RGL1 |
| miR-106b-5p | BEND3P3, DNM1P46, DNMBP-AS1, HYMAI, LINC00667, LINC00924, LINC01550, MBNL1-AS1, MEG3, MESTIT1, MIR99AHG, PWARSN, XIST, ZNF204P | ATP1A2, CYBRD1, NR4A3, PPP1R12B, SLC2A4 |
| miR-181b-5p | BEND3P3, GGTA1P, LINC00667, LINC01140, LINC01366, LOC100128164, LOC654342, XIST, ZNF192P1, ZNF204P, ZNF300P1 | ACVR1C, PDE5A, PPP1R12B, RYR3, S1PR1, THRB |
| miR-1301-3p | BEND3P3, CDC14C, EMX2OS, GOLGA8IP, LINC00667, LOC100128164, MBNL1-AS1, TRHDE-AS1, ULK4P3, ZNF192P1 | ANK2, CD34, RUNX1T1, SLC17A7 |
| miR-454-3p | MIR100HG, MIR99AHG, PWARSN, TPTE2P1, XIST | CAV2, CREB5, DPYSL2, EDN1, MET, PDE5A, RUNX1T1, TGFBR2 |
| miR-760 | CES1P1, CLEC4GP1, GOLGA6L5P, HAND2-AS1, LINC00284, LINC00341, LINC00982, LOC100128164, RAMP2-AS1, ZNF204P | FGF1, NGFR, SCN4B |
| miR-19a-3p | AGPAT4-IT1, CES1P1, GOLGA8IP, LINC00667, USP32P1 | CREB5, IGF1, NR3C2, NTRK2, SCN4B, SLC26A4, TFPI |
| miR-590-3p | HYMAI, LINC00472, LOC90768, MIR4697HG, XIST | CX3CL1, LPL, LRP1, SEMA3E, ST8SIA1, WASF3 |
| miR-141-3p | LINC00472, MBNL1-AS1 | ADRB1, CALCR, COL4A6, CSF3, IRS2, NFASC, NR3C1, THRB |
| miR-301a-3p | EMX2OS, HYMAI, MIR99AHG, PWARSN, XIST | ARHGEF4, NR3C2, PDE5A, PDGFRA, RUNX1T1, |
| miR-340-5p | LINC00889, LOC284578, XIST | ACVR1C, CAV2, DMD, FGF2, IGF1, NR3C1, RUNX1T1 |
| miR-15a-5p | SMIM10L2A, XIST | FOXO1, HPSE2, IGF1, MASP1, PAK7, SEMA3A, SEMA6D |
| miR-182-5p | LOC100128164, MST1P2, PGM5P2, SMIM10L2A | EPHB1, FGF9, MET, RECK, ST8SIA1 |
