# Supplementary figures and images for: Comprehensive Analysis of lncRNA and miRNA Regulatory Network Reveals Potential Prognostic Non-coding RNA Involved in Breast Cancer Progression
Source: Front Genet. 2021 Jun 18;12:621809. doi: 10.3389/fgene.2021.621809 (PMC8253500; doi:10.3389/fgene.2021.621809)

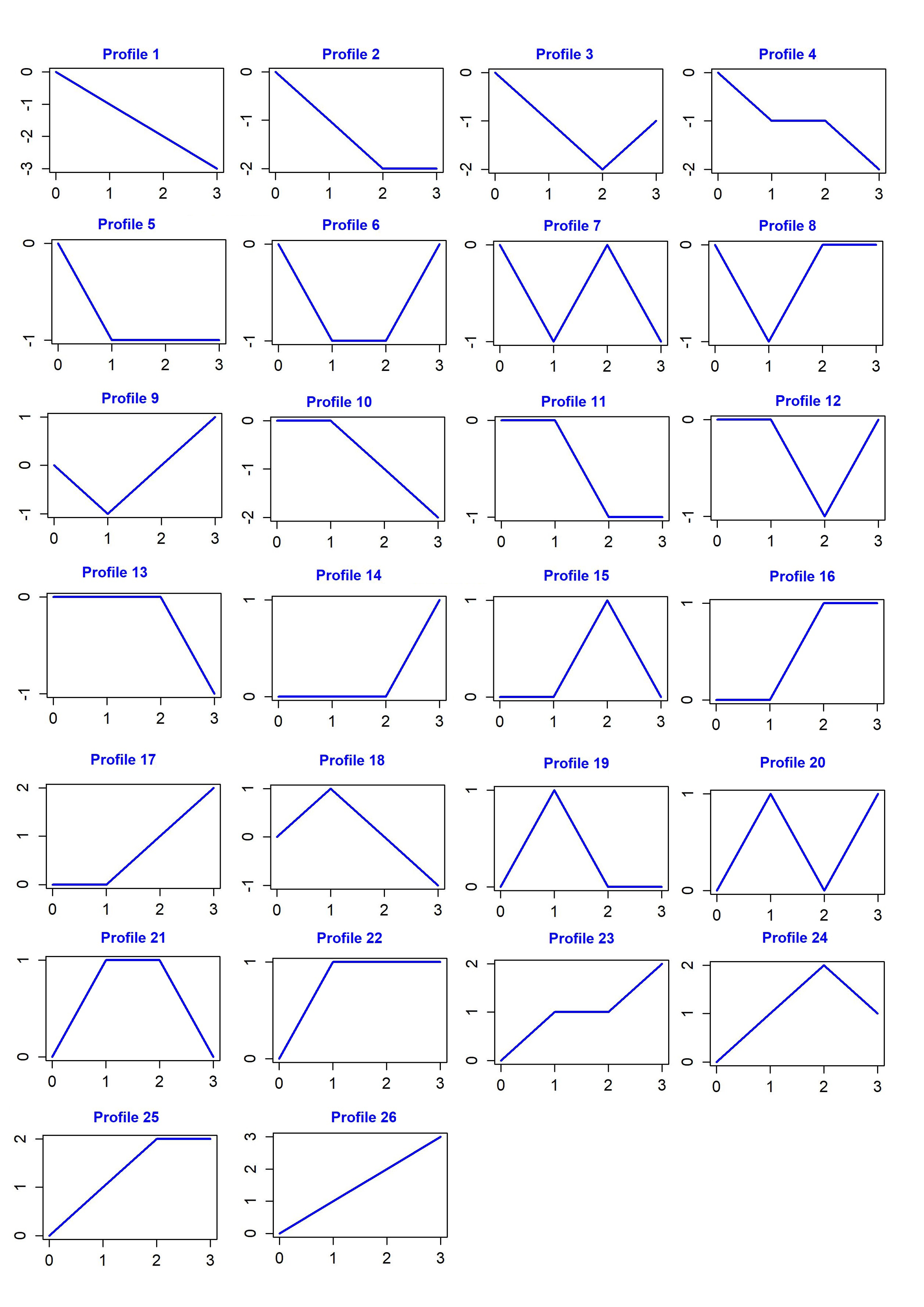

Supplement: Supplementary file 2 [file Image_1.TIF]

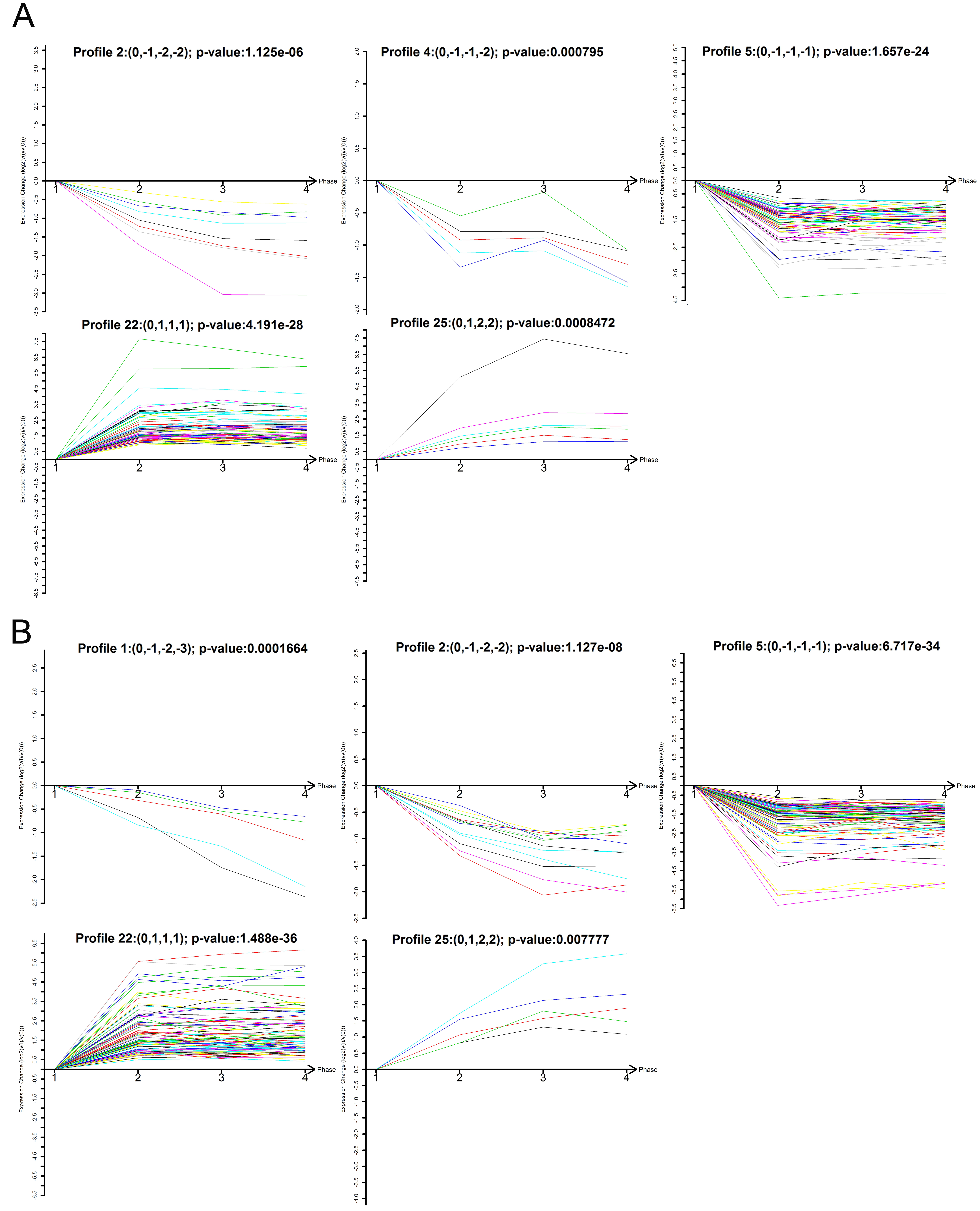

Supplement: Supplementary file 3 [file Image_2.TIF]

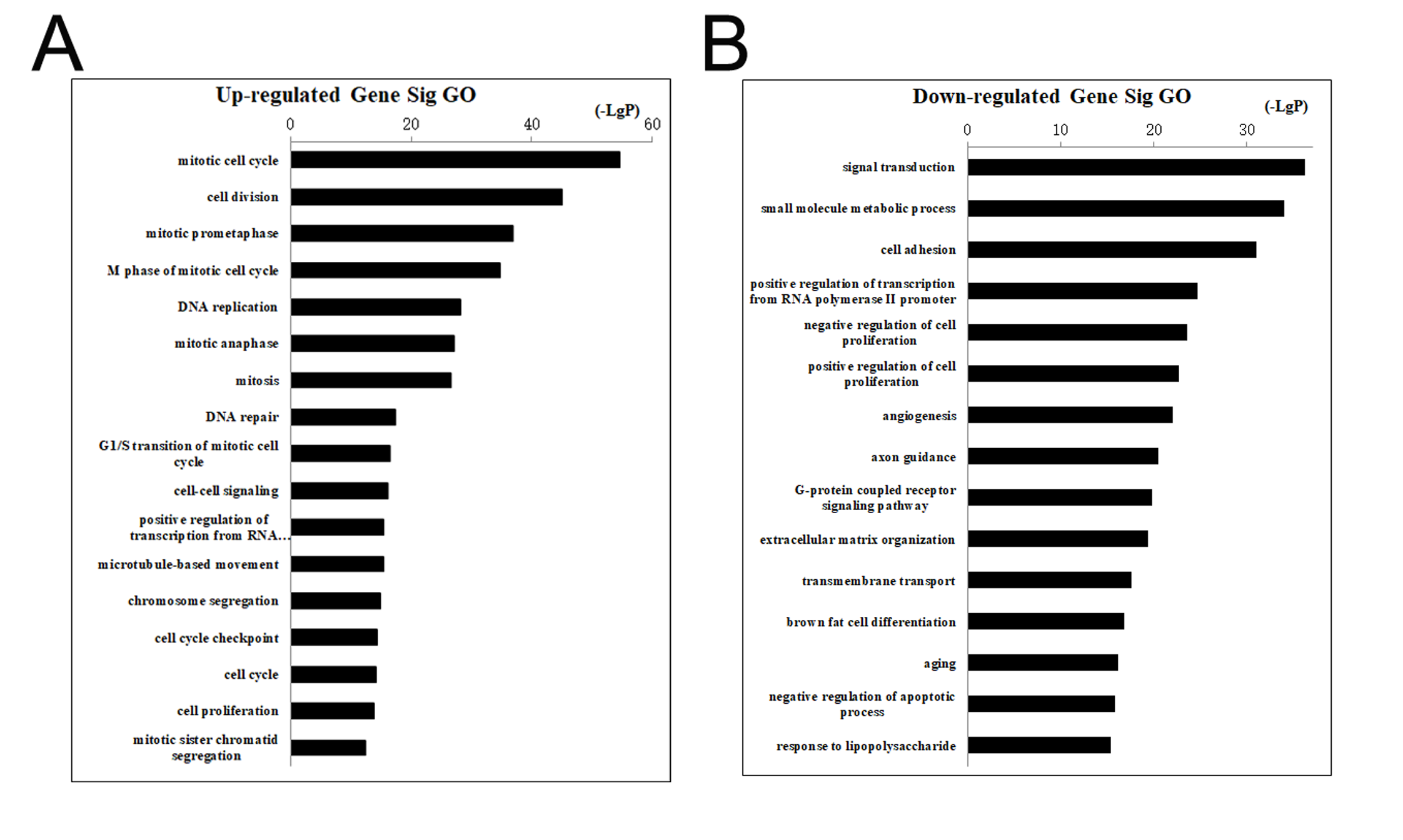

Supplement: Supplementary file 4 [file Image_3.TIF]

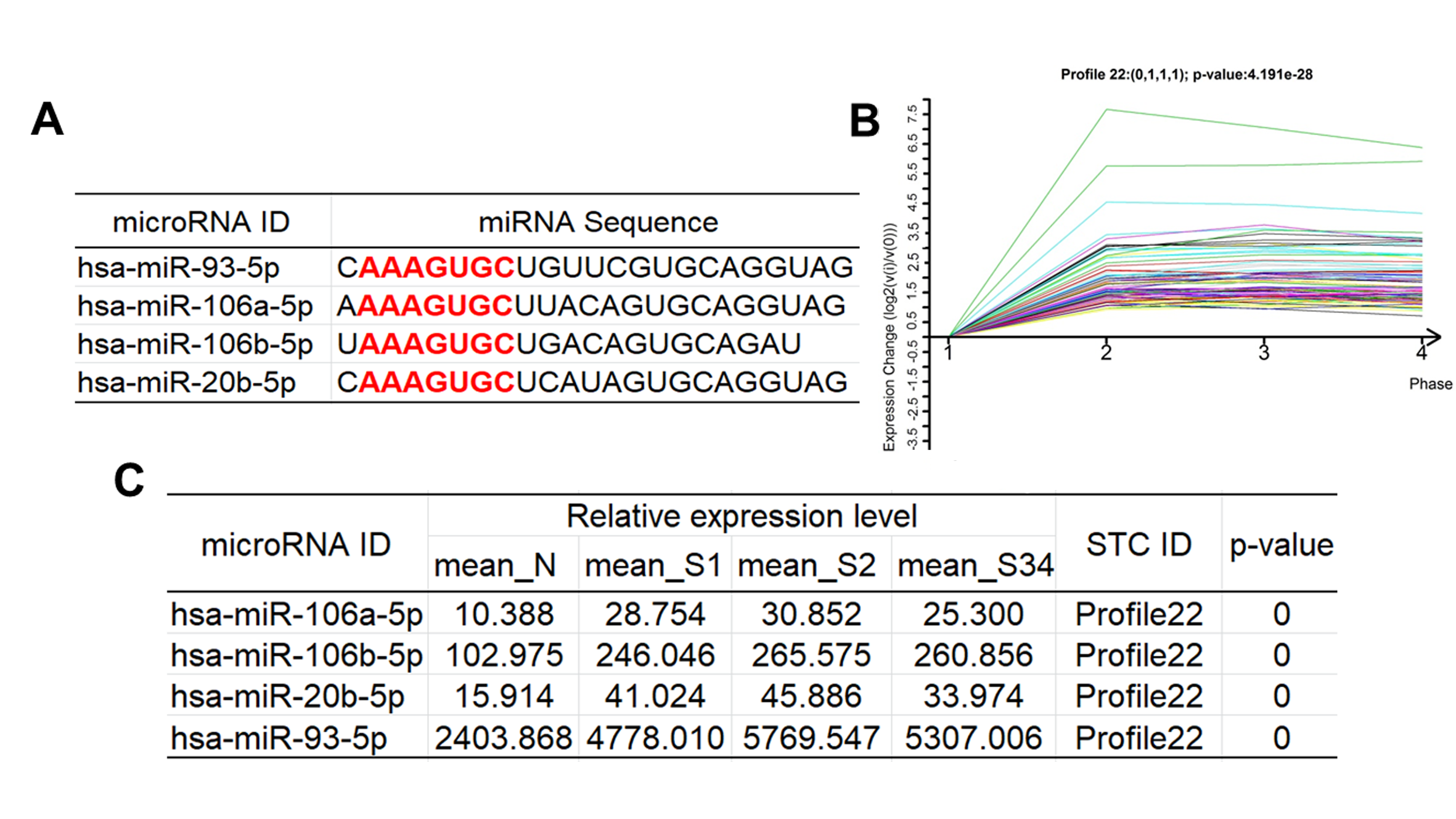

Supplement: Supplementary file 5 [file Image_4.TIF]

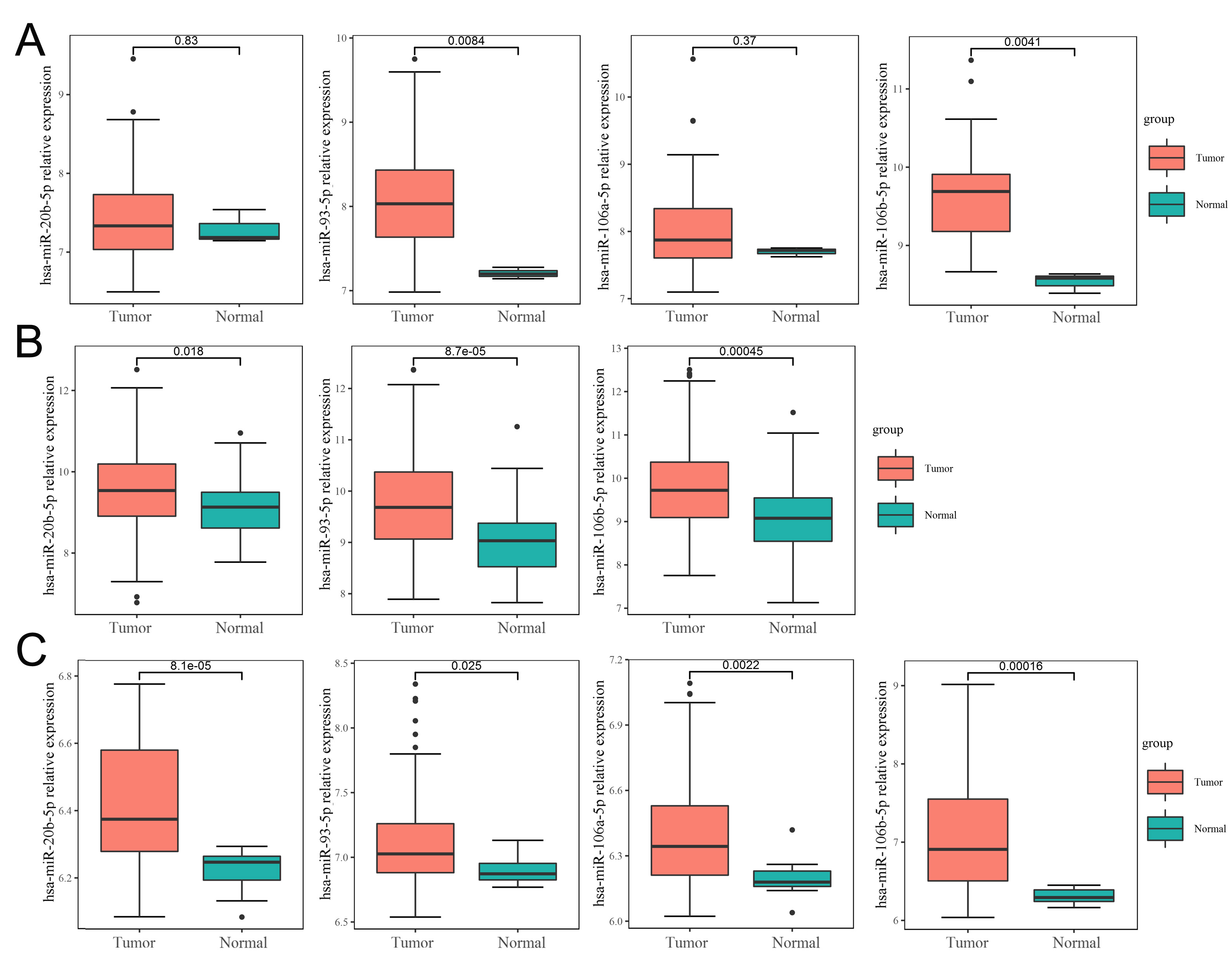

Supplement: Supplementary file 6 [file Image_5.TIF]
